# Elevated Major Adverse Cardiac Event (MACE) Risk With a HEART Score of 3: A Single-Site Retrospective Validation Study

**DOI:** 10.7759/cureus.83898

**Published:** 2025-05-11

**Authors:** David R Janese, Marshall Byun-Andersen, Danielle Handrop, Kelly Bihm, Sarah Bertrand, Payton Mangham, Marjan Trutschl, Phillip Kilgore, Urska Cvek, John Felty

**Affiliations:** 1 Emergency Medicine/Family Medicine, Louisiana State University Health Shreveport, Shreveport, USA; 2 Emergency Medicine, Louisiana State University Health Shreveport, Shreveport, USA; 3 Emergency Medicine, Louisiana State University Shreveport School of Medicine, Shreveport, USA; 4 Psychiatry, Louisiana State University Health Shreveport, Shreveport, USA; 5 Computer Science, Louisiana State University Shreveport, Shreveport, USA

**Keywords:** chest pain, clinical decision-making, heart score, myocardial infarction, risk-benefit assessment, troponin

## Abstract

Objective: The HEART pathway is a widely used clinical decision-making tool to risk-stratify patients presenting with chest pain in the emergency department (ED). A HEART score of 0-3 is generally accepted as “low risk,” often serving as a threshold for safe discharge. This study aimed to evaluate the performance of the HEART pathway in an urban academic ED and determine the associated rate of major adverse cardiac events (MACE) at a HEART score of 3. MACE were defined as all-cause mortality, cardiovascular death, myocardial infarction, heart failure hospitalization, or stroke within 90 days.

Methods: We conducted a retrospective chart review of 1,284 ED visits for chest pain from September 1, 2017, to August 31, 2018, at Louisiana State University Health Shreveport (LSUHS), Shreveport, Louisiana. HEART scores, demographics, and 90-day MACE outcomes were collected and analyzed using non-parametric statistical methods.

Results: Of 1,284 patients, 79 (6.2%) experienced MACE. Among patients with a HEART score of 3 or less, the MACE rate was 4.4% using Wilcoxon's rank-sum test (p < 0.001). The HEART score showed a positive correlation with MACE using Spearman’s rank correlation coefficient (ρ = 0.223, p < 0.001). Age correlated moderately with the HEART score (ρ = 0.568), but not with MACE. Gender showed no significant correlation with either HEART score or MACE outcomes.

Conclusion: A HEART score of 3 was associated with a MACE rate higher than the traditionally accepted 2% threshold for low risk. Clinicians should approach disposition decisions for these patients with caution and consider shared decision-making and observation. Further research is needed to refine HEART score interpretation at this critical threshold.

## Introduction

Chest pain is one of the most common chief complaints in the emergency department (ED), necessitating effective risk stratification to determine safe patient disposition. The HEART score was developed in 2008 as a clinical decision tool to identify low-, moderate-, and high-risk patients presenting with undifferentiated chest pain [1]. It incorporates five components, namely, history, electrocardiogram (ECG), age, risk factors, and troponin, each scored from 0 to 2, for a maximum score of 10. Based on the total score, patients are stratified into low- (0-3), moderate- (4-6), or high-risk (7-10) categories for major adverse cardiac events (MACE) [2]. MACE were defined as all-cause mortality, cardiovascular death, myocardial infarction, heart failure hospitalization, or stroke within 90 days.

Numerous studies have supported the utility of the HEART score for guiding discharge decisions and reducing unnecessary hospital admissions and testing [2,3]. However, recent evidence has questioned the safety of discharging patients with a HEART score of 3, with some studies reporting missed MACE rates as high as 3.3% in this group, exceeding the generally accepted 2% safety margin [3,4].

A potential explanation lies in the score’s subjectivity, particularly the “history” component, which depends on provider gestalt without clear criteria [5]. This variability may contribute to inconsistent scoring and missed risk. Alternative tools like the Emergency Department Assessment of Chest Pain Score (EDACS) attempt to address these concerns by introducing objective criteria for symptom evaluation [6].

In this context, the current study aimed to evaluate the HEART score at a single academic ED. The traditionally accepted threshold of ≤3 as “low risk” implies that such patients are often discharged without further testing or observation. However, exceeding a 2% MACE rate at this level is clinically meaningful, which challenges assumptions of safety, raises potential medicolegal risks if adverse events occur post-discharge, and may lead to suboptimal resource utilization by prematurely ending evaluation. Notably, the “history” component of the HEART score is subjective and provider-dependent, introducing variability that could affect local performance and reliability, especially at the critical decision point of a score of 3. Moreover, population-specific factors such as higher baseline cardiovascular risk, comorbidities, or socioeconomic disparities in an urban academic center may further influence the tool’s predictive accuracy. This study aimed to assess MACE outcomes at this commonly used threshold.

## Materials and methods

Study design and setting

This retrospective cohort study was conducted at Louisiana State University Health Shreveport (LSUHS), an urban academic medical center in Shreveport, Louisiana, with an annual ED census exceeding 65,000 patient visits. The study period encompassed ED encounters between September 1, 2017, and August 31, 2018. Institutional Review Board (IRB) approval was obtained prior to study initiation.

**Figure 1:**
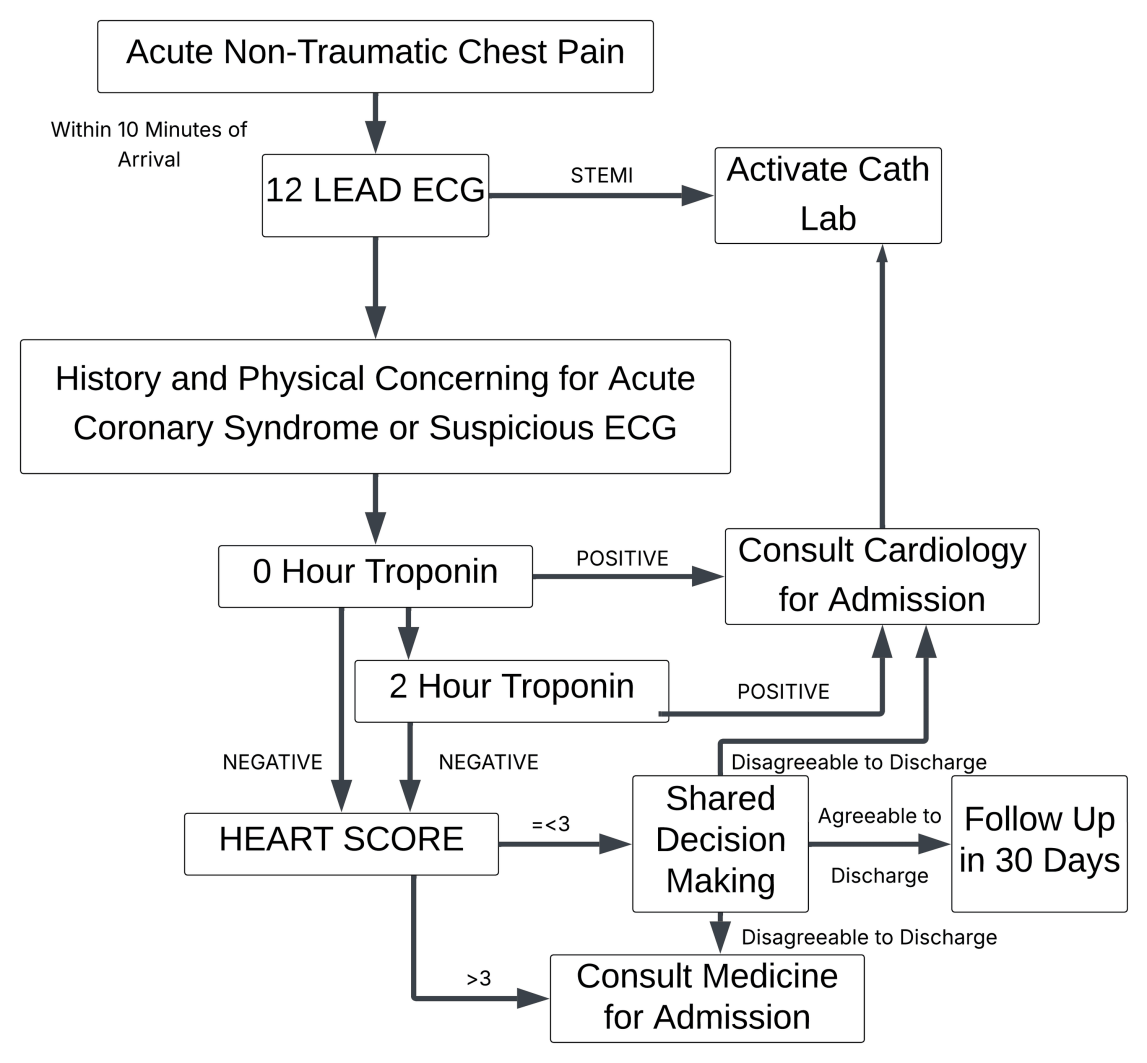
HEART pathway The image is under a Creative Commons License for free access. ECG: electrocardiogram, Cath Lab: Catheterization Laboratory

At the time of the study, the HEART pathway (Figure 1) and the HEART score were integrated into the electronic health record (EHR) system as a structured clinical prompt when troponin testing was ordered. Providers received a single formal educational session on HEART score application and interpretation as part of routine departmental training. No refresher sessions were conducted during the study period. 

Patient selection

Inclusions

Patients, aged 18 to 98 years, were included if they presented with chest pain or symptoms consistent with acute coronary syndrome (ACS), including exertional dyspnea, radiating discomfort, or unexplained epigastric pain. Symptom consistency with ACS was operationalized using predefined criteria adapted from institutional chest pain protocols; although no formal checklist was used, reviewers assessed documentation for classic features such as substernal pain, exertional onset, or relief with rest/nitroglycerin.

Exclusions

Patients were excluded if they had trauma, malignancy, or surgical emergencies (e.g., gastrointestinal perforation or acute abdomen), were undergoing dialysis or had end-stage renal disease, carried a diagnosis of systemic lupus erythematosus or other inflammatory/rheumatologic conditions, had isolated psychiatric or respiratory complaints not attributable to ACS (e.g., pneumonia, pneumothorax), left against medical advice or were not seen, or were incarcerated. These conditions were excluded because they may confound troponin levels or ECG interpretation, reflect non-cardiac etiologies of chest pain, or complicate outcome assessment. 

HEART score components and data collection

The HEART score comprises five components, namely, history, ECG, age, risk factors, and troponin, each scored from 0 to 2 based on defined clinical criteria (Table 1). Scores were auto-populated in the EHR if the provider completed the HEART pathway prompt. For encounters lacking documented HEART scores, trained reviewers retrospectively assigned scores using provider documentation and standardized definitions derived from institutional protocols and original HEART pathway publications. Definitions included classifying a “highly suspicious” history as chest pain that was substernal with radiation, exertional in onset, or relieved by nitroglycerin. Nonspecific ECG abnormalities encompassed T-wave inversions or ST-segment flattening. Recognized risk factors included hypertension, diabetes, hyperlipidemia, smoking, obesity, and known coronary artery disease.

**Table 1:** HEART score component criteria The image is under a Creative Commons License for free access. Reference: [1]

Component	0 points	One point	Two points
History	Slightly suspicious	Moderately suspicious	Highly suspicious
ECG	Normal	Nonspecific repolarization disturbances	Significant ST-depression or elevation
Age	<45 years	45–65 years	>65 years
Risk Factors	No risk factors	1–2 risk factors	≥3 risk factors or known atherosclerosis
Troponin	≤ normal limit	1–3× normal limit	>3× normal limit

Three reviewers (two board-certified emergency physicians and one senior medical student with research training) performed retrospective scoring. Discrepancies were resolved through group discussion and consensus, although formal interrater reliability (e.g., Cohen’s kappa) was not assessed due to resource limitations. Troponin levels were analyzed using the i-Stat point-of-care system, with normal and abnormal thresholds defined according to manufacturer specifications and HEART score guidance. Scoring thresholds were applied according to established HEART score criteria, relative to the manufacturer’s normal range [2].

MACE outcomes included all-cause mortality, cardiovascular-specific mortality, myocardial infarction, unplanned hospitalization for heart failure, and stroke or cerebrovascular events within 90 days of the index ED visit. These were identified through retrospective chart review and cross-referenced with a regional health information exchange to capture return visits or admissions at external institutions. All patients with incomplete HEART score components or insufficient follow-up data were excluded from the final analysis. No imputation methods were used.

Statistical analysis

All data were analyzed using R (R Foundation for Statistical Computing, Vienna, Austria). The distribution of HEART scores was assessed for normality using the Shapiro-Wilk test, which indicated a non-normal distribution (p < 0.001); thus, non-parametric statistical methods were applied throughout. The Wilcoxon's rank-sum test was used to compare median HEART scores between patients who did and did not experience a MACE. Correlation analyses were conducted using Spearman’s rank correlation coefficient (ρ) to evaluate the relationships between HEART score, age, and MACE occurrence. Gender associations with HEART score and MACE were assessed using the Kruskal-Wallis test. A p-value of <0.05 was considered statistically significant for all comparisons. Patients with missing HEART score components or without documented 90-day MACE outcomes were excluded from the analysis; no imputation methods were used. In addition, patients with incomplete provider documentation that precluded accurate retrospective HEART score assignment were also excluded. Although a formal a priori power analysis was not performed due to the retrospective nature of the study, the sample size (n = 1,284) and observed MACE rate suggest adequate statistical power to detect a ≥2% absolute difference in MACE occurrence between HEART score strata with an alpha of 0.05.

## Results

Cohort characteristics

A total of 1,284 patient encounters met inclusion criteria for analysis; the mean age was 51.38, the median age was 52, and the male/female ratio was 56%/44% (Table 2). Of these, 79 patients (6.2%) experienced MACE within 90 days of their ED visit. The remaining 1,205 patients (93.8%) did not experience MACE during the follow-up period.

**Table 2:** Clinical and demographic summary

Characteristic	Value
Total patients	1284
Mean age	51.38
Median age	52
Female (%)	56%
Male (%)	44%

The mean HEART score among the patients who experienced MACE was 5.43, compared to a mean score of 3.60 in patients without MACE (p < 0.001) (Table 3). 

**Table 3:** Statistical summary and correlations

Comparison	Result	p-value
MACE vs. non-MACE (mean score)	5.43 vs. 3.60	<0.001
HEART score vs. MACE	Spearman ρ = 0.223	<0.001
Age vs. HEART score	Spearman ρ = 0.568	<0.001
Age vs. MACE	Spearman ρ = 0.051	0.23
Gender vs. HEART score	No significant difference	0.123
Gender vs. MACE	No significant difference	0.432

HEART score distribution and normality

The distribution of HEART scores was non-normal, as assessed by the Shapiro-Wilk test (p < 0.001), justifying the use of non-parametric methods for subsequent analysis. 

 MACE at low HEART scores

Among the 478 patients with a HEART score of 3 or less, 21 (4.4%) experienced MACE. This value significantly exceeds the generally accepted 2% threshold for safe ED discharge in low-risk chest pain patients [3,4]. Notably, none of the patients with a HEART score of 0-2 experienced a MACE, suggesting a potential safety cutoff at that lower threshold. Conversely, patients with a HEART score of 10 all experienced a MACE, reinforcing the predictive value of higher scores. The mean HEART Score for no MACE was 3.6, whereas the confirmed MACE HEART score average was 5.43 (Table 3).

Correlation analyses

Spearman’s correlation coefficient (ρ) was used to evaluate the relationship between HEART score and MACE occurrence. A weak but statistically significant positive correlation was identified (ρ = 0.223, p < 0.001), indicating that increasing HEART scores were associated with a higher risk of MACE.

There was also a moderate positive correlation between age and HEART score (ρ = 0.568, p < 0.001), which was expected since age is one of the five components of the score. However, when age was analyzed in relation to MACE alone, the correlation was weak and not statistically significant (ρ = 0.051, p = 0.23), suggesting that age alone may not be a strong predictor of adverse cardiac events in this cohort (Table 3).

Gender did not demonstrate significant associations with either HEART score or MACE outcome. Specifically, the relationship between gender and HEART score was non-significant (p = 0.123), as was the relationship between gender and MACE occurrence (p = 0.432), based on the Kruskal-Wallis testing (Table 3).

## Discussion

In this single-center retrospective study, we observed a 90-day MACE rate of 4.4% among patients with a HEART score of 3, a finding that exceeds the widely accepted 2% threshold used to define low-risk chest pain cohorts appropriate for ED discharge [3,4]. The implications of this discrepancy are significant, as the HEART score is widely utilized in emergency medicine decision-making, and its low-risk cutoff has been commonly operationalized at scores ≤3 [1,2].

While previous authors such as Weinstock et al. and Than et al. have reported MACE rates near or above 3% in HEART score 3 patients, our findings align most closely with those studies and extend their implications by suggesting that a more conservative discharge threshold of ≤2 may better reflect true low-risk status (3,4]. Our results are consistent with earlier studies that found non-negligible MACE rates in patients classified as low risk by the HEART criteria. Mahler et al. reported a 1.7% MACE rate in low-risk HEART score patients, while Weinstock et al. described a missed MACE rate of up to 3.3% [2,3]. Than et al. further emphasized that many emergency clinicians consider a ≤2% miss rate acceptable when evaluating chest pain patients for discharge, reinforcing the significance of our findings [4]. Notably, none of the patients in our study with a HEART score of 0-2 experienced MACE, supporting this as a potentially safer threshold for early discharge. Conditional discharge pathways, such as early follow-up, outpatient stress testing, or shared decision-making, may be warranted when the HEART score is 3, particularly in higher-risk populations.

One likely contributor to this elevated MACE rate is the subjectivity inherent in the “history” component of the HEART score. This element relies heavily on provider interpretation of the patient’s symptom quality and does not have clearly defined objective criteria [5]. Brady and de Souza have pointed out that this subjectivity introduces variability and limits inter-provider reproducibility [5]. In fact, a validation work by Backus et al. identified notable interrater differences when scoring the history component, underscoring its inconsistency [7,8].

To address these concerns, other risk stratification tools such as the Emergency Department Assessment of Chest Pain Score (EDACS) have emerged. EDACS applies structured symptom criteria in place of subjective impressions, which may enhance scoring consistency [6]. Greenslade et al. demonstrated that EDACS performs comparably to HEART in identifying low-risk patients while reducing variability and subjectivity [9]. Herman and Weingart have also reviewed the two scores head-to-head, emphasizing EDACS’s potential advantage in reproducibility and clarity of use [6].

The timing of troponin testing may have also impacted predictive performance. Our institution utilized a two-step, 0-hour i-Stat troponin measurement and two-hour i-Stat Troponin measurement. While consistent with standard HEART implementation, this approach may miss delayed troponin rises, particularly in patients presenting early after symptom onset [1]. 

Another important factor affecting the accuracy of HEART scoring in our study was the type of troponin assay used. We employed the i-Stat point-of-care troponin I system, which is widely available and convenient, but lacks the analytical sensitivity of contemporary high-sensitivity cardiac troponin (hs-cTn) assays [8]. Carlton et al. showed that use of hs-cTn assays enables earlier identification of myocardial injury and supports discharge decisions after a single test in appropriately selected low-risk patients [7]. Twerenbold et al. likewise confirmed that hs-cTn assays outperform conventional assays in sensitivity, reducing the likelihood of missed myocardial infarction, particularly in the low- to intermediate-risk population [8].

Our study found no statistically significant association between gender and either HEART score or MACE outcome. While some prior research has identified sex-related differences in ACS presentation and outcomes, other large-scale studies have reported similar diagnostic performance of the HEART score in men and women [10]. Our findings support the latter perspective, suggesting that gender may not substantially influence HEART-based risk stratification in this setting.

The observation that age correlated strongly with HEART score but not with MACE suggests a potential disconnect between structural scoring and clinical risk. One plausible explanation is that age contributes linearly to the HEART score, but its true relationship with MACE may be nonlinear or modified by comorbidities and baseline health status. For example, an older patient with well-controlled risk factors may score higher without being at greater short-term cardiac risk. This suggests the need for refined age-weighting or adjusted risk models in future HEART score iterations.

Given that none of the patients in our cohort with a HEART score of 0-2 experienced a MACE, this subgroup appears to represent a truly low-risk population, appropriate for early ED discharge. By contrast, a score of 3 was associated with a significantly elevated event rate above the 2% safety threshold. These findings support the consideration of a revised low-risk threshold of ≤2 in clinical practice. Alternatively, patients with a HEART score of 3 may benefit from conditional discharge pathways, such as observation units, serial troponin testing, shared decision-making discussions, or expedited outpatient cardiology follow-up. Incorporating such strategies could help balance safety with efficient resource utilization while acknowledging the limitations of the HEART score at this intermediate threshold.

Limitations

This study has several limitations. First, it was conducted at a single academic center, which may limit generalizability to community hospitals or non-urban settings. The retrospective nature of the study introduces inherent bias, and data accuracy was dependent on the completeness and clarity of provider documentation.

In cases where the HEART score was not recorded in real time, retrospective assignment based on provider documentation may have introduced subjectivity. While scoring was conducted using the rubric (Table 1) by trained reviewers, we did not perform formal interrater reliability testing. Previous work has shown substantial variability in the “history” component between providers [5,11]. If bias occurred systematically (e.g., over- or under-categorizing historical features), it could affect event distribution across score strata. Future validation efforts should assess interrater agreement, especially for components prone to interpretation variability.

Furthermore, follow-up relied on institutional electronic health records and regional health information exchange data. While MACE outcomes were identified through chart review and a regional health information exchange, events occurring at out-of-network hospitals or outside the region could have been missed. Finally, our institution used point-of-care troponin testing rather than high-sensitivity assays. Prior studies have demonstrated that high-sensitivity troponin can detect myocardial injury earlier and with greater precision, which may improve risk stratification and reduce false negatives [7,9].

## Conclusions

In this single-center retrospective study, patients presenting to the ED with a HEART score of 3 experienced a 4.4% 90-day MACE rate, exceeding the widely accepted 2% threshold used to define a low-risk population. By contrast, no MACE events occurred among patients with HEART scores of 0-2, supporting the potential use of ≤2 as a safer discharge cutoff. These findings suggest that patients with a HEART score of 3 may benefit from extended observation, serial troponin testing, or shared decision-making strategies rather than automatic discharge. Future studies should validate this threshold prospectively and explore integration of high-sensitivity troponin assays and a combination of validated tools to refine early risk stratification.
